# Occurrence of multidrug-resistant extended-spectrum β-lactamase (ESBL)-producing *Escherichia coli* in fecal and environmental samples from Belgian livestock farms

**DOI:** 10.1128/spectrum.03591-25

**Published:** 2026-05-18

**Authors:** E. Lambrecht, M. Heyndrickx, G. Rasschaert

**Affiliations:** 1 ILVO, Flanders Research Institute for Agriculture, Fisheries and Food 74875 , Merelbeke-Melle, Belgium; National Institute of Science Education and Research, Odisha, India

**Keywords:** *Escherichia coli*, antibiotic resistance, resistance genes, manure, extended-spectrum β-lactamases

## Abstract

Antimicrobial resistance is a major global health concern, with extended-spectrum β-lactamase (ESBL)-producing bacteria posing a significant threat. Food production animals have been indicated as an important source of ESBL-producing bacteria. This study examined the occurrence of ESBL *Escherichia coli* in Belgian livestock (cattle, calves, pigs feces, and broiler ceca) and farm environments. Antimicrobial susceptibility of *E. coli* isolates (*n* = 1,219) was screened using microbroth dilution (EUVSEC), and *bla_TEM/SHV/CTX-M_* genes were further identified by PCR and sequencing. ESBL-producing *E. coli* isolates were more common in broiler samples (10% ceca, 2.5% environment) compared to those of pigs (1% feces, 2% environment), cattle (1% feces), and calves (1% feces). Among the 67 ESBL isolates, 63% carried *bla_CTX-M_* genes, and 55% *bla_TEM_* genes. *bla_SHV_* genes (13%) appeared only in isolates from broiler origin (ceca and farm environment). All ESBL-producing *E. coli* isolates were resistant to between 3 and 13 out of the 19 antimicrobials tested. In addition, a rapid PCR-based screening method in manure DNA extracts was evaluated for its ability to detect *bla_TEM/SHV/CTX-M_* genes compared to the conventional culture-based approach. These β-lactamase genes were detected in 98% of the samples (*bla_TEM_*: 95/101, *bla_CTX-M_*: 6/101, bla*_SHV_*: 0/101). The low prevalence of *bla_CTX-M_* and *bla_SHV_* in manure DNA extracts compared to the corresponding *E. coli* isolates may be due to PCR sensitivity issues or matrix inhibitory effects. The presence of ESBL-producing *E. coli* in broiler ceca, livestock feces, and manure raises concerns about the potential transmission of resistance genes in agriculture, highlighting the need for ongoing surveillance and responsible antibiotic use.

**IMPORTANCE:**

This research addresses the pressing global health concern of antimicrobial resistance, focusing on extended-spectrum β-lactamase (ESBL)-producing bacteria. The study investigates the occurrence and distribution of ESBL-producing *Escherichia coli* in Belgian livestock, including cattle, calves, pigs, and broiler chickens, as well as in their farm environments. The findings provide valuable data to inform farmers, veterinarians, and policymakers to raise awareness and support evidence-based antimicrobial stewardship.

## INTRODUCTION

The increase in antimicrobial resistance is a major global health concern, with extended-spectrum β-lactamase (ESBL)-producing bacterial pathogens emerging as a significant threat in both human and veterinary medicine. ESBLs are bacterial enzymes that inactivate β-lactam antibiotics, including penicillins, first, second, and third cephalosporins, and aztreonam, making them ineffective for treating infections (1). β-Lactam antibiotics are among the most frequently used worldwide (2), targeting a broad spectrum of both gram-positive and gram-negative bacterial pathogens.

Numerous ESBL enzyme variants have been identified to date, all of which, by definition, hydrolyze extended-spectrum β-lactam antibiotics and are inhibited by clavulanate. However, their encoding genes and substrate specificities vary considerably. ESBLs have been classified into 12 structural/evolutionary families by amino acid sequence similarity: TEM, SHV, CTX-M, OXY, IRT, CMT, GES, PER, VEB, BEL, TLA, and SFO. The most prevalent ESBL genes are *bla_TEM_*, *bla_SHV_*, and *bla_CTX-M_* (1), with hundreds of gene variants identified to date (https://card.mcmaster.ca/). Since not all gene variants confer an ESBL phenotype, accurate gene sequencing, and/or phenotypic antibiotic susceptibility testing is imperative. Phylogenetic analyses revealed that ESBL TEM variants are derived from a common broad-spectrum (non-ESBL) ancestor gene (TEM-1/2) by point mutations causing amino acid substitutions that enable the hydrolysis of expanded spectrum cephalosporins (1). This is the same for SHV variants (SHV-1 ancestor). By contrast, the CTX-M family constitutes a diverse, heterogeneous group. Based on amino acid sequence homologies, they are clustered into five main groups: CTX-M-1, CTX-M-2, CTX-M-8, CTX-M-9, and CTX-M-25. Recent studies indicate the existence of a sixth group, CTX-M-151 (3).

ESBL-encoding genes are often located on plasmids, transposons, or integrons, facilitating their spread within and between bacterial cells and species. ESBL genes are predominantly found in Enterobacterales (especially *E. coli*, *Klebsiella,* and *Salmonella*), but they are also detected in *Pseudomonas*, *Vibrio,* and *Aeromonas* species.

Bacterial isolates are identified as ESBL-producers through phenotypic testing, which detects enhanced susceptibility to third-generation cephalosporins when combined with β-lactamase inhibitors like clavulanic acid. In contrast, AmpC β-lactamase-producing isolates hydrolyze cephalosporins and cephamycins but are generally not inhibited by clavulanic acid. Their presence can mask ESBL activity, highlighting the importance of screening for both enzyme types to accurately assess β-lactam resistance (4).

The selective pressure exerted by (mis)use of antibiotics in animal production and human healthcare promotes rapid ESBL gene evolution and mobilization (5). ESBLs have become endemic worldwide. In the 90s, TEM and SHV ESBLs were the most identified ones, but since 2000, CTX-M enzymes have been recognized as the dominant ESBL type globally (6). Isolates carrying ESBL-encoding genes have been detected in e.g., nosocomial and community settings, soil, environmental waters, as well as livestock and food products (7, 8). Several correlations have been reported between ESBL*-*producing *E. coli* resistance profiles and sequence types from production animals (9–12) and human isolates (13). Their widespread nature and gene mobility make it difficult to track transmission pathways and directions, making a One Health approach crucial for effective surveillance, prevention, and control of ESBL-producing bacteria. The WHO recommends monitoring of ESBL*-*producing *E. coli* as an indicator of resistance development in gram-negative bacteria and as a relevant, representative proxy for the magnitude and trends of the global antimicrobial resistance problem (9). In the EU, monitoring of ESBL-producing *E. coli* is mandated in food-producing animals (feces) and certain food products (14). Routine monitoring of antimicrobial resistance in the environment, including manure used as fertilizer, is not yet implemented on a large scale. Manure and feces are regarded as a reservoir for antibiotic residues, resistance genes, and resistant bacteria. Its application as soil fertilizer (raw or treated) in agriculture may pose a risk of dissemination of resistance genes and resistant bacteria to crops and environmental soil and water (15–18).

The aim of this study was to determine (i) the occurrence of ESBL genes in *E. coli* isolates from ceca and feces of food-producing animals (broilers, pigs, cattle, and calves), manure, and the farm environment, and to assess (ii) their antimicrobial susceptibility patterns. In addition, a rapid PCR-based screening method in manure DNA extracts was included to quickly capture the presence of *bla_TEM/SHV/CTX-M_* genes independently of bacterial culturability. The PCR method was evaluated for its ability to detect *bla_TEM/SHV/CTX-M_* genes compared to the conventional culture-dependent, isolate-based approach.

## MATERIALS AND METHODS

### Antimicrobial susceptibility patterns of ESBL-producing *E. coli*

#### *E. coli* isolate selection

A selection of 1,219 *E. coli* isolates from livestock manure (*n* = 33), feces of pigs (*n* = 187), cattle (*n* = 194), and calves (*n* = 189), cecal content from broilers (*n* = 210), and swabs from the farm environment (*n* = 406), previously collected on Belgium farms and slaughterhouses from 2007 to 2022, was made (19–21). While some animal and environmental samples may originate from the same farms or time periods, no metadata were available to reliably link samples at the farm or temporal level. These isolates belong to the MB collection at ILVO and were stored in a ULT freezer. All isolates originated from screenings on RAPID’ *E. coli* (Bio-Rad), except for those from manure (*n* = 33), which were selected from RAPID’ *E. coli* with 0.25 mg/L cefotaxime (19). Antibiotic resistance profiles previously obtained by EUVSEC Sensititre microbroth dilution method were inspected. Briefly, bacterial colonies were resuspended in H_2_O (0.5 McFarland) and further suspended in cation-adjusted Mueller Hinton broth with TES buffer (Thermo Fisher) and inoculated onto an EUVSEC plate (EUVSEC I, Thermo Fisher Scientific), which contains dilutions of 14 lyophilized antibiotics. Minimum inhibitory concentrations were determined after incubation. Results were interpreted according to EUCAST epidemiological cutoff values. A multidrug-resistant isolate is one defined as resistant to at least one agent in ≥3 antimicrobial categories (22).

From the *E. coli* collection, 77 cefotaxime- and/or ceftazidime-resistant *E. coli* isolates (EUCAST ECOFF_FOT_ 0.25 mg/L, ECOFF_TAZ_ 0.5 mg/L) were retained for further ESBL testing.

#### Assessing ESBL and/or AmpC phenotypes

Confirmatory tests for ESBL production were performed by the microbroth dilution method with a second panel of antimicrobials (EUVSEC 2 plates, Thermo Fisher Scientific). ESBL phenotypes are identified by cefotaxime/ceftazidime resistance (R > 1 mg/L) and synergy with clavulanic acid (≥3 twofold concentration decrease in minimal inhibitory concentration by clavulanic acid). AmpC phenotypes were identified by resistance to cefoxitin (R > 8 mg/L).

#### Detection of *bla_TEM/SHV/CTX-M_* genes

Two complementary multiplex PCR assays (Monstein et al. and Dallenne et al.) were employed to maximize detection of β-lactamase genes. Rapid cell lysates were prepared from one colony in a total volume of 100 mL of distilled water (17 min 90°C), followed by a centrifugation step (1 min 10,000 × g) of the cell suspension. The supernatant was used in subsequent PCRs.

The presence of bla*_SHV_*, bla*_CTX-M_*, and *bla_TEM_* genes was detected by a multiplex PCR according to Monstein et al. (23). Briefly, the PCR mix contained 1× buffer II, 1.5 mM MgCl_2_ (Thermo Fisher Scientific), 2U AmpliTaq polymerase (Applied Biosystems), 200 µM dNTPs (Thermo Fisher Scientific), 0.4 µM of each primer (bla-SHV.SE/AS, TEM-164.SE/AS, and CTX-M-U1/U2) and 2 µL cell lysate in a total volume of 25 µL. The reaction parameters were: 15 min denaturation at 95°C, followed by 30 cycles at 94°C for 30 s, 60°C for 30 s, and 72°C for 2 min, and a final extension at 72°C for 10 min. The amplification of resistance genes, *bla*_SHV_: 747 bp, *bla*_CTX-M_: 593 bp, and *bla*_TEM_: 445 bp, was verified by 1.5% agarose electrophoresis.

The multiplex PCRs developed by Dallenne et al. (24) were used to detect the presence of *bla_TEM_* and *bla_SHV_*, and classify *bla_CTX-M_* genes into major groups (i.e., group 1, 2, 9, 8, and 25). The setup consisted of three multiplex PCRs. For each multiplex, the PCR mix contained 1× buffer II, 1.5 mM MgCl_2_, 1U AmpliTaq polymerase, 200 µM dNTPs, 0.4 µM of each primer (except for multiCTXMGp1-2_rev and Gp2_for: 0.2 µM), and 2 µL lysate in a total volume of 50 µL. The reaction parameters were: 10 min denaturation at 94°C, followed by 30 cycles at 94°C for 40 s, 60°C for 40 s, and 72°C for 1 min, and a final extension at 72°C for 7 min. The PCR amplicons (*bla_TEM_*: 800 bp, *bla*_SHV_: 713 bp, *bla_CTX-M_1_*: 688 bp, *bla*_CTXM_2_: 404 bp, *bla_CTX-M_9_*: 561 bp, *bla_CTX-M_8/25_*: 326 bp) were separated by electrophoresis as described above. It should be noted that not all *bla_TEM_* and *bla*_SHV_ genes encode ESBLs. The PCRs used detect *bla_TEM_* and *bla*_SHV_ in general but do not differentiate between ESBL-producing and non-ESBL-producing alleles.

Therefore, once the presence of *bla_TEM/_ bla*_SHV/_*bla_CTX-M_* genes was confirmed, simplex PCR reactions with respective primers were performed, and amplicons were sequenced (Sanger, Macrogen) and mapped against the Comprehensive Antibiotic Resistance Database (https://card.mcmaster.ca/) to identify the exact ESBL gene variant.

### Culture-independent screening of manure samples

101 manure pit samples (calves and pigs), previously collected and frozen at −80°C (19), were used for the next experiments. Manure samples were plated on Brilliance ESBL *E. coli* (Oxoid) to enumerate ESBL-producing *E. coli* and ESBL coliforms. For each coliform colony morphology, one representative colony was selected and further identified by MALDI-TOF MS (microflexTM LRF mass spectrometer) according to the manufacturer’s instructions (Bruker Daltonics, Belgium). In parallel to the manure plating, DNA was extracted with the PowerSoil kit (Qiagen) and stored at −20°C.

Presence of *bla_TEM/SHV/CTX-M_* genes in manure DNA extracts was assessed by multiplex PCRs according to references 23, 24, as described above. To increase sensitivity and to reduce potential inhibiting matrix effects, for each sample, the simplex PCR variants (same concentrations and amplification parameters) were also tested, as well as diluting the DNA extracts 1/10 and 1/100 and the addition of 0.1% and 0.01% Triton-X100 (Merck) to the PCR mix.

### Data analysis

Exploratory data analysis, visualization, and statistical analyses were performed in R version 4.5.0. Insufficient data/time (2007–2022) prevented robust stratification by year. The data sets were therefore merged to preserve analytical consistency.

Associations between isolate origin (broiler, pig, calf, and cattle) and ESBL prevalence, or antibiotic resistance profiles, or detected resistance genes were evaluated using Fisher’s exact test, with adjusted (Bonferroni) *P* values < 0.05 considered statistically significant.

## RESULTS

### Antimicrobial susceptibility patterns of ESBL*-*producing *E. coli* isolates from manure, feces, and farm environment

*E. coli* isolates from manure (calves/pigs), broiler ceca, livestock feces (pig/cattle/calves), and farm environmental swabs were pre-screened for cefotaxime and/or ceftazidime resistance (Table 1); 77/1,186 isolates were classified as resistant according to EUCAST ECOFF breakpoints. Subsequent resistance testing using EUVSEC2 plates demonstrated that 60/77 *E. coli* isolates exhibited an ESBL phenotype (FOT or TAZ >1 mg/L and synergy with clavulanic acid), 8 a pure AmpC phenotype (FOX >8 mg/L, no synergy), and 7 a combined ESBL + AmpC phenotype. ESBL-producing *E. coli* were found in all tested matrices. Focusing on *E. coli* isolated from RAPID’ *E. coli* without selective cefotaxime (excl. manure isolates), ESBLs were significantly more prevalent (adjusted *P* value < 0.001, Fisher’s exact test) in broiler ceca (10%, 22/210) than in feces of pigs (1%, 2/187), cattle (1%, 2/194), and calves (1%, 2/189) and environmental swabs.

**TABLE 1:** Occurrence of ESBL, AmpC, and ESBL + AmpC phenotypes in cefotaxime and/or ceftazidime-resistant (CEFO/TAZ R) *E. coli* isolates from different origins^*b*^

	Manure			Ceca	Feces			Environment		Total
	Calves	Pig	P/C	Broiler	Pig	Cattle	Calves	Broiler	Pig	
CEFO/TAZ resistant isolates	5 (100^*a*^)	24 (100^*a*^)	4 (100^*a*^)	23 (11)	2 (1)	4 (2)	4 (2)	6 (3)	5 (2,4)	77
ESBL	3 (60^*a*^)	21 (88^*a*^)	2 (50^*a*^)	20 (9,5)	2 (1)	2 (1)	1 (0,5)	5 (2,5)	4 (2)	60
AmpC	0	1 (4^*a*^)	1 (25^*a*^)	1 (0,5)	0	2 (1)	1 (0,5)	1 (0,5)	1 (0,5)	8
ESBL + AmpC	2 (40^*a*^)	2 (8^*a*^)	0	2 (0,9)	0	0	1 (0,5)	0	0	7
Total number of isolates	5^*a*^	24^*a*^	4^*a*^	210	187	194	189	200	206	1,219

^*a*^ Selective isolation on cefotaxime agar plates; hence, the total number of isolates equals that of CEFO/TAZ R isolates p/c = either pig or calves.

^*b*^ Between brackets: relative occurrence = % of total number of isolates. Total number of isolates = all *E*. *coli* isolates incl. non-resistant CEFO/TAZ isolates.

ESBL-positive isolates from the broiler environments (2.5%, 5/200) originated from the drain gutter (*n* = 3), drinking system (*n* = 1), and a floor crack (*n* = 1). One pure AmpC-positive isolate was found in the drinking system. In the pig farm environment, ESBL-positive *E. coli* (2%, 4/206) were detected on the wall (*n* = 1), water pipe (*n* = 1), drinking system (*n* = 1), and drinking nipple (*n* = 1), and one AmpC-positive on the floor.

**Fig 1:**
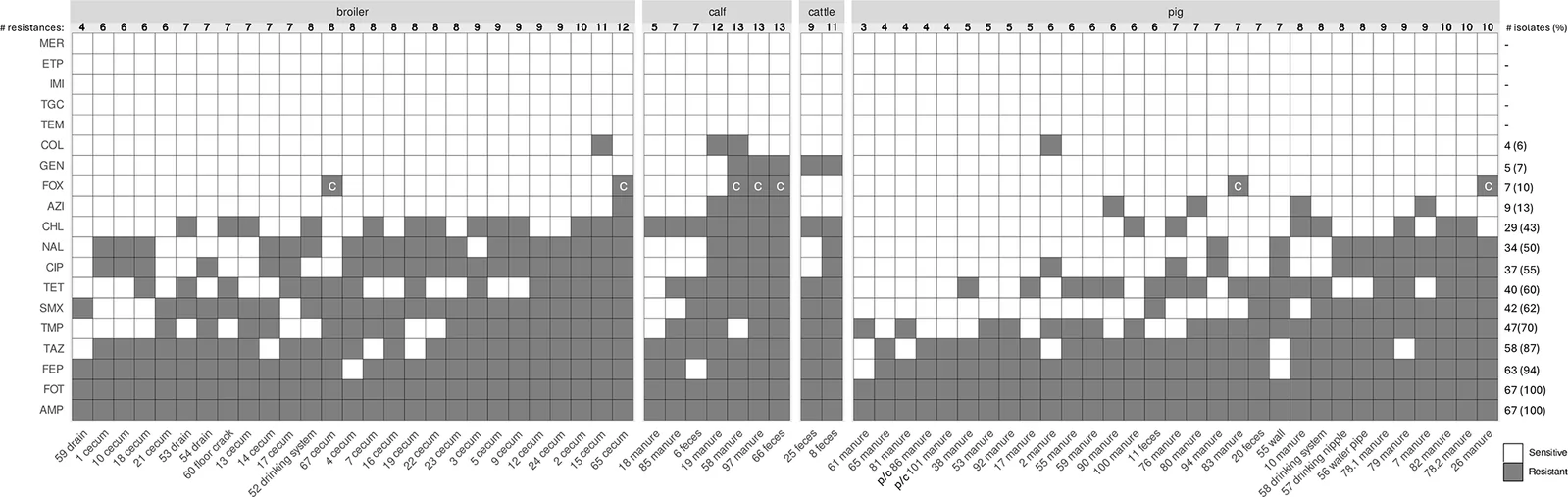
Antibiotic resistance profiles of ESBL*-*producing *E. coli* isolates. Sensitivity/resistance assessed by microbroth dilution (EUVSEC) and EUCAST ECOFF values. The numbers on top of the columns are total number of detected resistances per isolate, numbers at the row ends are the numbers of resistant isolates, with % in brackets (*n* = 67). C: ESBL + AmpC phenotype MER: meropenem, ETP: ertapenem, IMI: imipenem, TGC: tigecycline, TEM: temocillin, COL: colistin, GEN: gentamicin, FOX: cefoxitin, AZI: azithromycin, CHL: chloramphenicol, NAL: nalidixic acid, CIP: ciprofloxacin, TET: tetracycline, SMX: sulfamethoxazole, TMP: trimethoprim, TAZ: ceftazidime, FEP: cefepime, FOT: cefotaxime, AMP: ampicillin.

All ESBL-producing *E. coli* showed resistance to multiple antibiotics, ranging from a minimum of 3 to a maximum of 13 of the 19 tested antimicrobials (Fig. 1). A variety (*n* = 47) of resistance profiles was observed. No significant association was observed between antibiotic resistance profiles and isolate origin (Fisher’s exact test). All were resistant to cefotaxime and ampicillin, but none of them were resistant to tigecycline, carbapenems (imipenem, meropenem, and ertapenem), or temocillin. A total of 64 out of 67 isolates met the definition of multidrug-resistant. In contrast, pig manure isolates 65, 86, and 101 were resistant to only two antimicrobial categories (penicillins and 3rd/4th-generation cephalosporins).

### Genotyping/classification of ESBL-producing isolates

**Fig 2:**
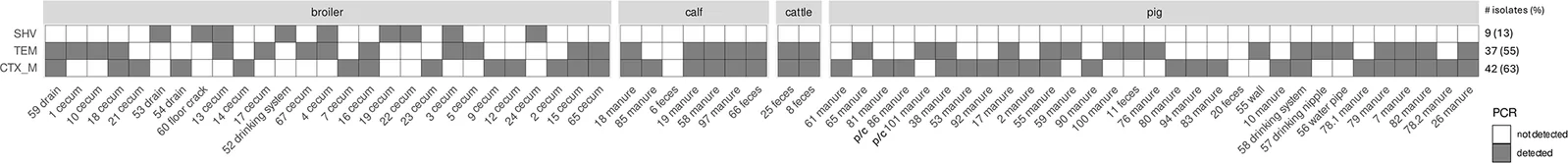
Presence of ESBL genes (*bla*_TEM,CTX-M, and/or SHV_) in *E. coli* isolates with ESBL phenotype. Numbers at the row ends are number of positive isolates, with % in brackets (*n* = 67).

The ESBL phenotype was confirmed by PCR in all but two of the 67 isolates (6_feces_calf and 20_feces_pig), demonstrating the presence of *bla_TEM_* (37/67, 55%), *bla_CTX-M_* (42/67, 63%), and/or *bla_SHV_* genes (9/67, 13%) (Fig. 2). Some isolates contained multiple β-lactamases: *bla_TEM_* in combination with *bla_CTX-M_* (20/67) or with *bla_SHV_* (2/67). Fisher’s exact test indicated there was no association between *E. coli* origin (animal and matrix) and occurrence of *bla_TEM_* or *bla_CTX-M_*. However, a significant association was found between animal and *bla_SHV_* prevalence (*P* = 0.001), as *bla_SHV_* genes were only detected in isolates from the poultry sector.

Sequencing of PCR amplicons revealed that CTX-M group 1 type 1 was predominant (*n* = 27/67, 37%). Other CTX-M types detected included CTX-M-15 (*n* = 1), CTX-M-32 (*n* = 3), CTX-M-1/61/138 (*n* = 9), CTX-M group 2 (*n* = 1), and group 9 (*n* = 1) genes. The TEM variants were diverse and consisted of TEM-1 (*n* = 16), TEM-15 (*n* = 5), TEM-57 (*n* = 1), TEM-84 (*n* = 2), TEM-135 (*n* = 1), TEM-225 (*n* = 2), and TEM-24/23 (*n* = 15). SHV genes included SHV-2 (*n* = 3), SHV-12 (*n* = 5), and SHV-8 (*n* = 8).

### Culture (in)dependent detection of *bla_TEM/SHV/CTX-M_* in manure samples

DNA extracts from 101 manure pit samples were screened for the presence of β-lactamase genes (*bla_TEM/SHV/CTX-M_*) by the multiplex PCRs of Monstein (20) and Dallenne (21). Overall, 99/101 manure samples tested positive for the presence of at least one of these genes. The majority of these samples tested positive for *bla_TEM_* (*n* = 95/101), while *bla_CTX-M_* was detected in 4/101 samples. *bla_SHV_* was not detected in any of the manure samples.

Differences were observed in the detection of TEM genes between the two multiplex PCR protocols, with the PCR described by Monstein (23) showing greater sensitivity for *bla_TEM_* genes (91/101 samples) in manure DNA extracts than the protocol by Dallenne (24) (55/101), although a combination of both PCRs showed the highest sensitivity. The simplex PCR variants i.e., including only the TEM primer pairs in the PCR, resulted in 95/101 (Monstein) and 82/101 (Dallenne) positive samples. Adding Triton-X-100 or diluting (1/10 and 1/100) the DNA extract never resulted in better detection of β-lactamase genes. The simplex PCR variant of Monstein detected, however, more *bla_CTX-M_* genes (6/101) than the multiplex one (4/101) and the simplex/multiplex according to Dallenne (both 2/101).

Direct plating of the corresponding manure samples (M&M) on ESBL *E. coli* selective agar identified only 29 out of the 101 samples as positive for cultivable ESBL-producing *E. coli,* with concentrations ranging from 1.30 log CFU/g up to 3.40 log CFU/g. The resistance profiles of those isolates can be seen in Fig. 2 (calf and pig manure samples). The majority of these manure isolates harbored *bla_CTX-M_* genes, but these could not be detected in the corresponding manure DNA extracts. In these 29 samples, other cultivable bacteria with ESBL phenotype were also detected (26/29 samples, min 1.30–max 3.42 log CFU/g). Those had a green colony morphology and were identified by MALDI-TOF as *Klebsiella, Enterobacter, and Serratia* species.

## DISCUSSION

Livestock have been indicated as an important source of ESBL-producing bacteria and a contributor to antimicrobial resistance in human pathogens (25). This study focused on the detection and characterization of ESBL-producing *E. coli* in Belgian livestock manure, feces, ceca, and farm environment. *Escherichia coli* are regarded as indicator organisms that provide information on the reservoirs of resistant bacteria that could potentially be transferred between animal populations and humans. They also provide indirect information on the reservoirs of resistance genes that could be transferred to bacteria that are pathogenic to humans and/or animals.

Antimicrobial susceptibility testing on *E. coli* isolates demonstrated that ESBL-producing *E. coli* were significantly more prevalent in broiler ceca (10%, 22/210) than in feces of pigs (1%, 2/187), cattle (1%, 2/194), and calves (1%, 2/189). This aligns with previous Belgian non-selective monitoring studies (2015–2023 [26]), which reported similar rates and, in analogy to other European countries, a higher prevalence in broiler ceca.

Among the farm environmental swabs, the current study also observed a slightly higher ESBL-producing *E. coli* prevalence in broiler farms (2.5%, 5/200) compared to those of pig farms (2%, 4/206). This is in agreement with other studies identifying the broiler production industry as the one with the highest prevalence compared to other animal sectors (27–29). This might be related to the high use of aminopenicillins during the short broiler lifespan (30), which may select for ESBL resistance. In addition, group treatment including sick and healthy animals via, e.g., drinking water, is common in broilers as opposed to individual antibiotic treatments with cattle and pigs; the former may exert a higher selection pressure and increase the development, selection, and spread of resistance (31).

In total, 63% of the ESBL-producing *E. coli* isolates harbored *bla_CTX-M_* genes, and in 47% of them, additional *bla_TEM_* genes were detected. CTX-M is regarded as the dominant ESBL family of ESBLs in human and animal strains (7) and has largely replaced TEM as the most prevalent ESBL type (32). Nonetheless, a large percentage of isolates (55%) in this study still contained *bla_TEM_* genes either exclusively (30%) or in combination with *bla_CTX-M_ (25%*). Harboring a variety in β-lactamase resistance genes offers a broader resistance spectrum, which can have a selective advantage. Since some resistance genes confer overlapping resistance profiles, this can favor resistance gene exchange and evolution through mutations (33). Sequencing revealed that CTX-M group 1 was the dominant CTX-M type; this is consistent with previous studies (34). *Bla_SHV_* genes (SHV-2 and SHV-12) were only detected in isolates from broiler origin. These specific SHV types are frequently encountered in ESBL *E. coli* (35), with SHV-12 being the most widespread variant in European countries in the poultry sector (29).

Two isolates (one pig feces and one calf feces) exhibited a clear ESBL phenotype but tested negative for *bla_CTX-M_*, *bla_SHV_*, and *bla_TEM_*. This might be explained by, for example, mutations in primer binding sites or the presence of less common ESBL genes (eg. GES, VEB, PER).

ESBL genes are often located on mobile elements such as plasmids, which also carry genes encoding resistance to other drug classes (1). This leads to multiresistant *E. coli,* which makes treatment challenging. In the current study, all ESBL-producing *E. coli* isolates were resistant to a minimum of 3 and a maximum of 13 tested antibiotics. Multiple co-resistance profiles could be observed, with many of them being observed only once in this study, indicating a diverse, dynamic resistance gene repertoire. As expected by definition, all ESBL-producing *E. coli* isolates were resistant to ampicillin and cefotaxime, and the majority were resistant to ceftazidime (87%), cefepime (94%, 4th-generation cephalosporin), and ciprofloxacin (55%). Resistance to these last four antibiotics and to nalidixic acid (50%), colistin (6%), and azithromycin (13%) is especially worrisome. These antibiotics are categorized by the WHO as highest priority critically important antimicrobials (36) and should be used with care, as they are often one of the last-resort treatments against multiresistant bacteria in human medicine. Seven isolates (cecum: 2, calf manure: 3, pig manure: 2) were also resistant to cefoxitin, indicating the presence of AmpC cephalosporinases, which broaden their resistance potential. In this study, two *E. coli* isolates from calf manure were resistant to all critical antibiotics, with one also showing resistance to cefoxitin, which is deeply concerning. Calf farms are often criticized for their antibiotic use. Compared to other sectors, they have some of the highest BD100 values (percentage of days an individual animal or group of animals in a farm is treated with antibiotics) (26). Occurrence of antibiotic-resistant *E. coli* and antibiotic residues have been shown to be higher in calf slurry than in cattle slurry manure (37).

Apart from analyzing *E. coli* isolates from ceca, feces, and the environment, this study also investigated the presence of *bla*_TEM_/*bla*_SHV_/*bla*_CTX_M_ genes in manure samples by comparing isolate-based, culture-dependent testing and faster culture-independent screening of manure DNA extracts. ESBL prevalence in cefotaxime-resistant *E. coli* isolates from calf and pig manure was high: 100% and 95%, respectively. It should be pointed out that a selective isolation protocol (0.25 mg/L cefotaxime) was used for manure ESBL-producing *E. coli* isolates. Therefore, prevalence levels of manure isolates should not be compared to those of environmental, feces, and cecum isolates, which were based on non-selective monitoring.

Screening of blaTEM/blaSHV/blaCTX genes by PCR might allow fast detection of resistance genes present in the broader bacterial population (*E. coli* and other organisms), which may not be recovered by standard culturing. Results of the fast multiplex and simplex PCR on manure DNA extracts revealed that almost all samples tested positive for TEM genes, as expected based on high TEM prevalence in the *E. coli* isolates. It should be noted that the general PCRs will also detect non-ESBL TEM genes (eg., TEM_1). Discrepancies between detected genes in DNA extracts from manure samples and those from corresponding *E. coli* isolates can be due to (i) the presence of non-*E*. *coli* ESBL species or (ii) diverse, multiple *E. coli* strains per sample, since only one ESBL*-producing E. coli* colony was selected for PCR. The simplex PCR protocol variants consistently demonstrated improved detection of *bla_TEM_* genes in manure. Exact sensitivity values could not be calculated, as the true number of positive (*bla_CTX-M/SHV/TEM_*) samples is unknown. The difference might be due to PCR inhibitors which might interfere more strongly in the complex multiplex reactions, where multiple primer sets are present. Remarkably, few samples tested positive for *bla_CTX-M_*, whereas this gene was dominant in the *E. coli* isolates from manure samples. This suggests that the applied *bla_CTX-M_* PCRs (multiplex/simplex) are not optimal yet for direct manure screening. In addition to the previously mentioned potential matrix inhibition, the suboptimal PCR results may be due to insufficient sensitivity to detect very low concentrations of *bla_CTX-M_* in manure.

Published data on ESBLs in manure are scarce, and prevalence rates in livestock vary widely by country and farm management. Comparison is further hampered by differences in methodology and isolation years. The ESBL *E. coli* prevalences we observed in cattle manure are in line with those reported by national monitoring studies in cattle feces (~60% from 2015 to 2023 [26]). By contrast, those of ESBL-producing *E. coli* in pig manure are higher than the rates reported for pig feces. Belgian monitoring studies report a decreasing trend in pig feces (63% in 2015 to 30% in 2023), mainly attributed to sensitization and efforts to reduce the use of antibiotics (26).

Livestock manure is used as organic fertilizer for crops and grasslands and can introduce antibiotic residues, resistance genes, and resistant bacteria into soil, groundwater, and crops (38). Several studies detected ESBL *E. coli* in fertilized soil (39–43) or reported a temporary increase in resistance genes in soil after fertilization (44, 45). In Belgium, around two-thirds of manure is applied untreated to agricultural fields (46). In contrast, European guidelines recommend treating manure before field application to avoid nitrate leaching (47). Such treatment may lower the abundance of antibiotic resistance genes (48)

### Conclusions

This study highlights the presence of ESBL-producing *E. coli* in Belgian manure, feces, and ceca from production animals and farm environments and supports antimicrobial resistance concerns within the One Health framework. The findings confirm a higher prevalence of ESBL *E. coli* in broiler ceca and farm environment isolates compared to those of pigs and cattle feces, in accordance with previous national and European studies. All ESBL-producing *E. coli* isolates were resistant to a minimum of 3 and a maximum of 13 (out of 19) tested antibiotics. There were various co-resistance profiles evident, indicative of the diverse, dynamic resistance gene pool.

The existing multiplex PCR protocols now in use for *bla*_TEM_/*bla*_SHV_/*bla*_CTX_M_ detection have not yet been refined for screening direct DNA extracts from manure. The presence of ESBL *E. coli* in livestock feces and manure raises concerns about the potential transmission of resistance genes through agricultural practices and emphasizes the need for continued surveillance, responsible antibiotic use, and improved manure management strategies to mitigate the spread of resistance.
